# Unexpected absence of fetal hemoglobin induction by lenalidomide in a patient with sickle cell anemia with concurrent multiple myeloma

**DOI:** 10.1186/s13023-025-04052-0

**Published:** 2025-10-17

**Authors:** Rasmus Rønnemoes, Jens Helby, Amina Nardo-Marino, Morten Hanefeld Dziegiel, Jesper Petersen, Agoston Gyula Szabo, Nina Toft, Andreas Glenthøj

**Affiliations:** 1 Department of Hematology, Copenhagen University Hospital - Rigshospitalet, Copenhagen, Denmark; 2 Department of Clinical Medicine, University of Copenhagen, Copenhagen, Denmark; 3 Department of Clinical Immunology, Copenhagen University Hospital – Rigshospitalet, Copenhagen, Denmark

## Abstract

Sickle cell anemia (SCA) and multiple myeloma (MM) are debilitating hematologic diseases impacting hemoglobin and plasma cells, respectively. Despite differing origins, they share overlapping clinical features including bone pain and anemia. A cornerstone of SCA is to induce fetal hemoglobin through hydroxyurea treatment. Lenalidomide, a central treatment of MM, has also been shown to induce fetal hemoglobin in vitro. Here we present a case of a young woman with SCA and subsequently multiple myeloma. When lenalidomide was used as part of multiple myeloma treatment, we investigated the proposed induction of fetal hemoglobin, using high-pressure liquid chromatography. Over a treatment period of 114 days, we did not observe lenalidomide-induced increases in fetal hemoglobin and were thereby unable to replicate in vitro findings. Moreover, the case highlights many difficulties of diagnosing and treating patients with SCA and concurrent malignancies.

**Supplementary Information:**

The online version contains supplementary material available at 10.1186/s13023-025-04052-0.

## Introduction

Sickle cell anemia (SCA) is a debilitating monogenic condition arising from homozygosity for the hemoglobin S (HbS) variant causing hemoglobin polymerization, rigid red blood cells (RBCs), and multi-organ damage [1].

The cornerstone of current treatment is hydroxyurea (HU), which primarily works by inducing fetal hemoglobin (HbF), which displaces HbS and inhibits polymerization [2]. Several novel strategies for inducing HbF are under investigation [3, 4]. Previous studies have hinted that lenalidomide, an immunomodulatory drug used for treatment of multiple myeloma, can potently induce HbF and has synergistic effects with HU [5].

In this report, we describe a case of a 36-year-old female with SCA undergoing multiple myeloma treatment with lenalidomide to investigate HbF induction and RBC health.

## Case presentation

A 36-year-old woman with SCA was admitted with severe lower back-pain and anemia (hemoglobin 3.7 g/dL). She had given birth two months prior and was actively breastfeeding. HU, which had previously increased her HbF levels to approximately 30%, had been discontinued for nearly two years due to pregnancy and breastfeeding. Ten days prior to admission, she had presented with her first vaso-occlusive event (VOE) in six years for which she had been shortly admitted.

A columnal X-ray revealed a collapsed vertebra Th11. This was treated conservatively with analgesics. Blood tests indicated Coombs positive hemolysis, which prompted prednisolone treatment for 3 days. This was discontinued when the Coombs-positivity was correlated to FyA antibodies developed after transfusions received during the recent admission. She was referred to endocrinological assessment for osteoporosis. A dual-energy X-ray absorptiometry-scan, which detected lumbar osteoporosis (T-score − 3,3), and routine osteoporosis blood-screening were performed. The overall conclusion was osteoporosis related to pregnancy and SCA. Treatment with teriparatide was initiated.

Two months later, worsening of back pain prompted a follow-up X-ray, which showed deterioration of the collapsed vertebrae leading to application of a 3-point corset.

During the following period, the patient engaged in physiotherapy, experiencing varying but tolerable pain levels. However, 10 months later she was readmitted with severe worsening of back pain, together with hypercalcemia and pneumonia. A new X-ray revealed a minor collapse of Th9.

A reexamination of the osteoporosis blood-screening taken 10 months earlier revealed an unnoticed IgA M-protein of 28 g/L. This prompted diagnostic work-up for multiple myeloma. At this point, the IgA M-protein was 30 g/L and serum Free Light Chain ratio was 238 with 1500 mg/L kappa-light chains. Bone-marrow biopsy confirmed the diagnosis of multiple myeloma with IgA isotype, and 60–70% infiltration of kappa-monoclonal plasma-cells.

Treatment with teriparatide was discontinued, and prednisolone initiated. Vertebroplasty of Th9 and Th11 was performed. One-week post-surgery, multiple myeloma treatment consisting of four series of Cyclophosphamide, Bortezomib, and Dexamethasone (CVD) was initiated. Transfusions were restricted by prior alloimmunization (Anti-c, -E, -Fya, -Jka, -S).

Multiple complications occurred during treatment, including dehydration, osteomyelitis, tongue swelling and, notably, severe VOE episodes following the initiation of each series. The latter requiring hospital admissions with extensive opioid treatment. After the fourth series of CVD daily morphine use exceeded 250 mg leading to somnolence. The patient further developed severe pulmonary hypertension and was admitted to the Intensive Care Unit (ICU). Manual exchange transfusion with 4 units of packed RBCs was performed during the 8-day ICU stay, and her condition gradually improved. Nonetheless, she was markedly physically weakened after her month-long admission.

Following CVD treatment, the M-protein was immeasurable and kappa-light-chain slightly elevated at 35.9 mg/L (normal range 6.7-22-4 mg/L), at this point treatment with high-dose-melphalan with autologous stem cell transplantation (HDM-ASCT) was planned.

Due to SCA, the prior stem cell mobilization was performed solely by plerixafor [6] over the course of three days.

HDM-ASCT supported by automated exchange transfusion with highly matched RBCs was uncomplicated. During treatment-free follow-up, regular automated exchange transfusions were performed, and she was additionally surgically treated for bilateral osteonecrosis of the hip.

1.5 years following HDM-ASCT, the patient experienced a relapse of multiple myeloma with an increase in serum M-protein and serum kappa free light-chains. A PET-CT-scan revealed progressive bone disease in the pelvis. The bone marrow remained hypocellular with no evidence of clonal plasma cell infiltration.

Relapse treatment consisted of re-induction and new stem cell collection with the aim of performing a second HDM-ASCT. Treatment was stepwise with first increasing doses of lenalidomide monotherapy starting at daily 12,5 mg, then in the third cycle, dexamethasone was added and from the fourth to sixth cycles, daratumumab was added.

**Fig. 1:**
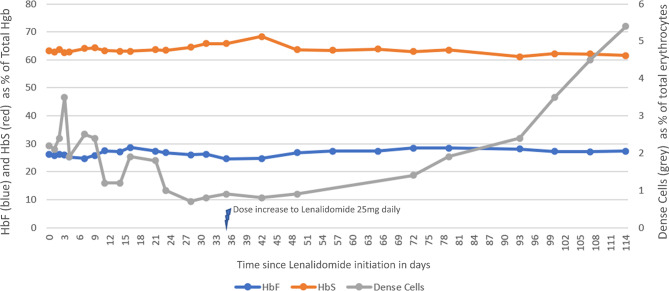
Levels of HbF, HbS and dense cells during the first four cycles of treatment with lenalidomide (with increasing dosage, subsequent Dexamethasone addition and Daratumumab added for the final recorded series)

**Fig. 2:**
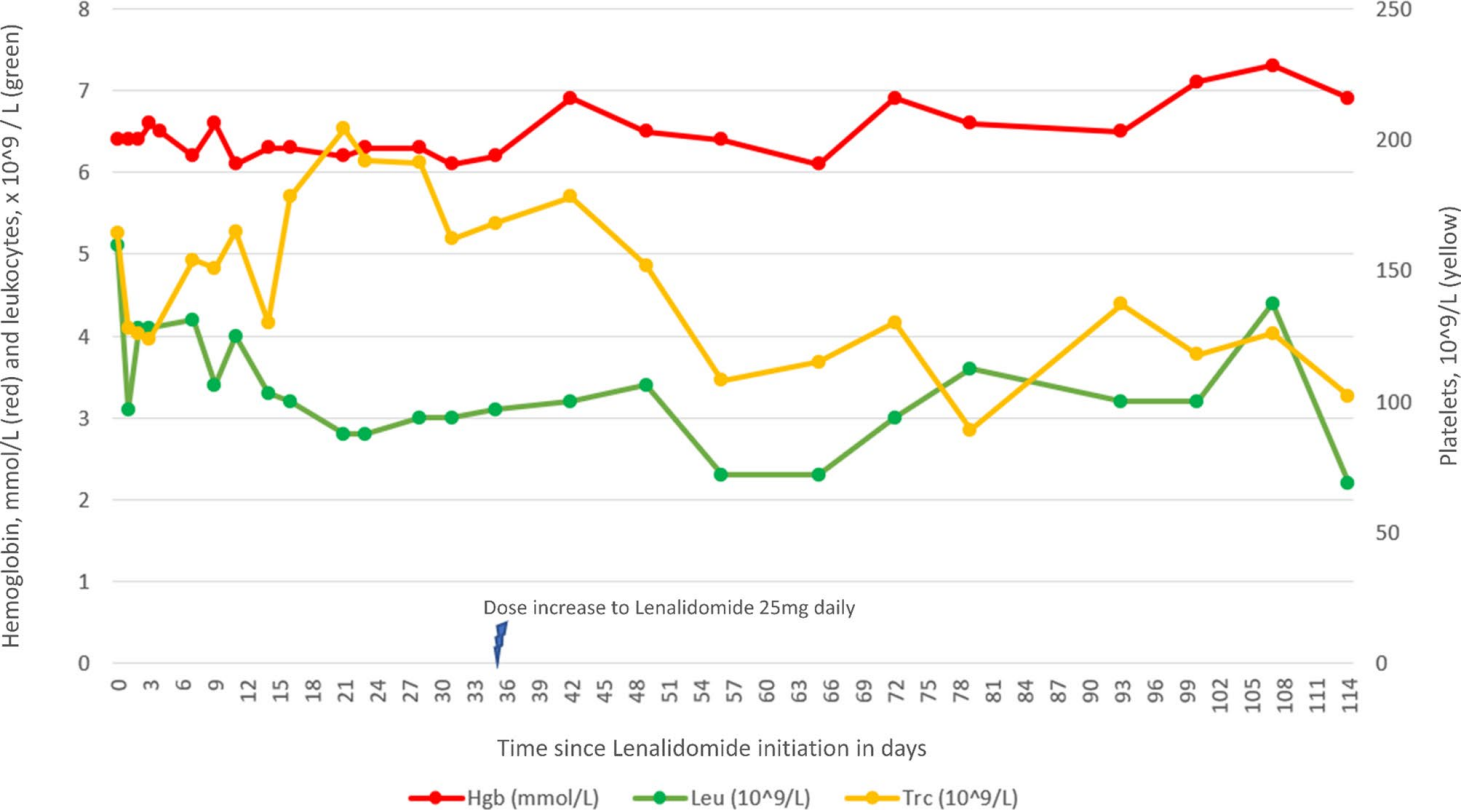
Levels of hemoglobin, leukocytes, and platelets during the first four cycles of treatment with lenalidomide with increasing dosage, subsequent Dexamethasone addition and Daratumumab added for the final recorded series)

During this treatment series containing lenalidomide, we evaluated the proposed induction of HbF by high-pressure liquid chromatography (HPLC) as previously described [7], by flow cytometry using a BD FACSCanto II instrument, and by dense cells measurement (MCHC >41 g/dl) using a Siemens Advia 2120i hematology analyzer (Fig. 1) [8]. No effect on HbF levels was observed during the first four cycles (in total 114 days) of lenalidomide. Flow cytometry showed a consistently high proportion of HbF-containing RBCs (84.0–91.5%) that remained overall stable during treatment (Supplementary Table S1). After increasing the dose of lenalidomide, there was a steady rise number of dense cells, a negative disease marker indicating the presence of irreversibly sickled cells and poorer outcomes [9]. Notably, this increase did not coincide with any pain episodes. Furthermore, the patient did not experience any significant cytopenias despite the increased dosing (Fig. 2).

The patient achieved very good partial response as a result of the re-induction. Subsequently, stem-cell collection was unsuccessfully attempted twice with high-dose cyclophosphamide and plerixafor. Finally, hydroxyurea was discontinued for two weeks and a new attempt at plerixafor stimulation yielded enough stem cells to conduct a second HDM-ASCT. The second transplant was performed following an automated exchange transfusion and proceeded without significant complications. The patient achieved very good partial response. Following HDM-ASCT, lenalidomide maintenance was initiated but discontinued after two months due to cytopenia.

One year later, the patient experienced her second multiple myeloma relapse with increasing serum kappa free light-chains and a PET-CT-scan revealing multiple new skeletal lesions for which she received radiotherapy. Two series of daratumumab monotherapy were followed by addition of lenalidomide and dexamethasone, with dose-reduction due to cytopenia. The patient achieved partial response. Bortezomib was later added to the treatment regimen, which remains ongoing with unchanged response.

## Discussion

This case highlights the difficulties of treating patients with SCA and concurrent malignancies. Potential complications from or interactions with standard treatments may necessitate vastly different approaches to the choice and dosage of chemotherapy, stem-cell mobilization, and blood transfusions.

We further had the opportunity to study the proposed effect of lenalidomide on HbF induction in a 36-year-old patient with SCA and multiple myeloma. We could not in vivo replicate the positive effect previously reported in vitro [5] despite successful HbF induction by HU in the same patient, previously. A probable cause for this could be differing mechanistic pathways for HbF induction by these drugs, HU through activation of soluble guanylyl cyclase [10] and inhibition of ribonucleotide reductase [11], and immunomodulatory drugs (IMiDs) such as Lenalidomide through inhibition of γ-globulin regulators such as *BCL11A* [12].

The patient had elevated baseline HbF value of 18.2% (Supplementary Table S1) which increased while on HU to a maximum value 28.8%. The patient’s elevated baseline HbF could theoretically be caused by polymorphisms in *BCL11A* [13], which might explain why lenalidomide did not increase HbF further, as this pathway would already be affected. This is speculative, as there are currently no known polymorphisms blocking the interaction of lenalidomide and the *BCL11A* promoter, and no sequencing of the patient was performed.

Following the second HDM-ASCT, HU treatment was discontinued due to cytopenia. Interestingly, the patient’s HbF level remained elevated (Supplementary Table S1). While the underlying mechanism is uncertain, it is conceivable that the autologous transplant process here favored engraftment of hematopoietic stem cells with high HbF production [14].

Finally, the case serves as a reminder that patients may have concurrent hematologic diseases with common denominators such as anemia and bone pain. This may delay correct diagnosis and treatment. As SCA treatment options improve and the lifespan for individuals living with SCA lengthens, we can expect an increasing number of SCA patients with concurrent malignant diagnoses, prompting research into the unique complications that may arise in these cases.

## Supplementary Information

Below is the link to the electronic supplementary material.

Supplementary Material 1

## Data Availability

The data that support the findings of this study are available from the corresponding author upon reasonable request.
