# Supplementary material for: Unexpected absence of fetal hemoglobin induction by lenalidomide in a patient with sickle cell anemia with concurrent multiple myeloma
Source: Orphanet J Rare Dis. 2025 Oct 17;20:522. doi: 10.1186/s13023-025-04052-0 (PMC12534949; doi:10.1186/s13023-025-04052-0)
Supplement: Supplementary file 1 — Supplementary Material 1 [file 13023_2025_4052_MOESM1_ESM.docx]

Title: Unexpected Absence of Fetal Hemoglobin Induction by Lenalidomide in a Patient with Sickle Cell Anemia with Concurrent Multiple Myeloma

Rasmus Rønnemoes, Jens Helby, Amina Nardo-Marino, Morten Hanefeld Dziegiel, Jesper Petersen, Agoston Gyula Szabo, Nina Toft, Andreas Glenthøj

# Supplementary material

**Supplementary Table S1: HbF fraction measurements and key events.**

| Day | HbF (%) | Key event | HbF RBC (%) |
| --- | --- | --- | --- |
| Day -1285 | 18.2 | First HbF measurement after pregnancy off HU treatment |  |
| Day -1285 | 2.3 | RBCX |  |
| Day -1284 | 2.4 |  |  |
| Day -1274 | 4.0 |  |  |
| Day -1273 |  | Start HU 1g/day |  |
| Day -1210 |  | Pause HU (neutropenia, thrombocytopenia) |  |
| Day -888 |  | Start HU 1g/day |  |
| Day -863 | 14.2 |  |  |
| Day -852 |  | Pause HU (myeloma diagnosis) |  |
| Day -833 |  | Start CVD (myeloma treatment) |  |
| Day -784 | 15.3 |  |  |
| Day -766 | 12.7 |  |  |
| Day -735 | 5.3 |  |  |
| Day -726 | 5.4 |  |  |
| Day -721 | 4.5 |  |  |
| Day -717 | 10.0 |  |  |
| Day -714 | 12.1 |  |  |
| Day -713 | 2.4 | RBCX |  |
| Day -700 | 9.1 |  |  |
| Day -691 | 12.1 |  |  |
| Day -685 | 12.1 |  |  |
| Day -685 | 12.4 |  |  |
| Day -683 | 4.5 | RBCX |  |
| Day -672 | 5.1 |  |  |
| Day -658 | 5.1 |  |  |
| Day -656 | 6.3 |  |  |
| Day -654 | 1.9 | RBCX |  |
| Day -648 |  | 1. ASCT |  |
| Day -632 | 5.7 |  |  |
| Day -623 | 2.2 | RBCX |  |
| Day -601 | 9.9 |  |  |
| Day -599 | 14.7 |  |  |
| Day -598 | 2.6 | RBCX |  |
| Day -538 | 14.7 |  |  |
| Day -526 | 16.9 |  |  |
| Day -524 |  | Start HU 500mg /day |  |
| Day -518 | 14.4 |  |  |
| Day -516 | 8.2 | RBCX |  |
| Day -515 | 10.4 |  |  |
| Day -514 | 7.7 |  |  |
| Day -513 | 8.0 |  |  |
| Day -510 | 8.3 |  |  |
| Day -510 | 5.1 | RBCX |  |
| Day -496 |  | Increase HU to 1g/day |  |
| Day -482 | 14.5 |  |  |
| Day -468 | 19.0 |  |  |
| Day -454 | 23.5 | Reduce HU to 500mg /day (leukopenia, thrombocytopenia) |  |
| Day -440 | 28.1 |  |  |
| Day -410 | 28.2 |  |  |
| Day -403 | 28.8 |  |  |
| Day -398 | 7.4 | RBCX |  |
| Day -335 | 17.8 |  |  |
| Day -284 | 23.2 |  |  |
| Day -235 | 26.4 |  |  |
| Day -196 | 26.0 |  |  |
| Day -194 | 6.1 | RBCX |  |
| Day -77 | 28.5 |  |  |
| Day 0 | 26.2 | Start Lenalidomide 10mg/day 3 weeks on, Pause HU | 90.4 |
| Day 1 | 25.8 |  | 90.5 |
| Day 2 | 26.2 |  | 91.4 |
| Day 3 | 25.9 | Resume HU 500mg/day | 91.5 |
| Day 4 | 25.3 |  |  |
| Day 7 | 24.7 |  |  |
| Day 9 | 25.8 |  | 89.8 |
| Day 11 | 27.5 |  |  |
| Day 14 | 27.1 |  |  |
| Day 16 | 28.7 |  | 89.6 |
| Day 18 | 28.6 |  |  |
| Day 21 | 27.3 |  | 88.6 |
| Day 23 | 26.8 |  |  |
| Day 25 | 26.1 |  |  |
| Day 28 | 26.0 |  |  |
| Day 30 |  |  | 86.1 |
| Day 31 | 26.2 |  |  |
| Day 35 | 24.6 | Increase Lenalidomide 25mg /day 3 weeks on | 85.4 |
| Day 42 | 24.7 |  | 84.0 |
| Day 49 | 26.8 |  | 84.7 |
| Day 56 | 27.4 |  | 86.8 |
| Day 65 | 27.3 | Add Dexamethasone (LenDex) 4mg d0, 10mg d8, 20mg d15, d22, d23 |  |
| Day 72 | 28.4 |  | 87.9 |
| Day 79 | 28.4 |  | 88.3 |
| Day 86 | 27.9 |  | 89.7 |
| Day 93 | 28.1 |  | 89.1 |
| Day 94 |  | Add Daratumumab (DaraLenDex) Reduced Lenalidomide 25mg/day 2 weeks on |  |
| Day 101 | 27.2 |  | 88.9 |
| Day 108 | 27.1 |  |  |
| Day 115 | 27.3 |  |  |
| Day 154 |  |  | 90.7 |
| Day 182 |  | Pause HU (stem cell harvest for 2nd ASCT) |  |
| Day 193 | 23.1 |  | 85.1 |
| Day 196 | 24.1 |  |  |
| Day 196 | <1.0 | RBCX |  |
| Day 199 |  | Start HU 500mg/day |  |
| Day 266 | 21.2 |  |  |
| Day 267 |  | Pause HU (2nd ASCT) |  |
| Day 268 | 5.4 | RBCX |  |
| Day 274 |  | 2nd ASCT |  |
| Day 308 |  | Start HU 500mg/day |  |
| Day 365 |  | Pause HU (cytopenia) |  |
| Day 399 | 27.5 |  |  |
| Day 668 | 24.9 |  |  |
| Day 798 |  | Start Lenalidomide 5mg/day 3 weeks on |  |
| Day 753 | 23.1 |  |  |
| Day 1358 | 21.9 |  |  |

**Supplementary Table S1: Measurements of HbF fraction and the proportion of red blood cells containing HbF (HbF RBC) in relation to key events.** Days presented are relative to the initiation date of Lenalidomide treatment (Day 0). Key events presented are those expected or suspected to influence the HbF fraction, Hydroxyurea (HU) treatment, Red blood cell exchange (RBCX), Cyclophosphamide, Bortezomib and Dexamethasone treatment (CVD), high-dose-melphalan with autologous stem-cell transplantation (HDM-ASCT) and Lenalidomide treatment.
